# CO_2_ ‐Dependent Promotion of Photosynthesis Drives Metabolic Photoacclimation in *Chlamydomonas reinhardtii*

**DOI:** 10.1111/ppl.70461

**Published:** 2025-08-27

**Authors:** Ana Pfleger, Erwann Arc, Thomas Roach

**Affiliations:** ^1^ Department of Botany University of Innsbruck Innsbruck Austria

**Keywords:** carbon, *Chlamydomonas reinhardtii*, metabolism, nitrogen, photoacclimation, photoinhibition, photosynthesis

## Abstract

Light and inorganic carbon (C_i_) drive photosynthesis, which fuels cellular maintenance, energy storage, and growth in photosynthetic organisms. Despite its pivotal role, how primary metabolism adjusts to contrasting light and C_i_ availability in algae remains elusive. Here, we characterized bioenergetics and profiled primary metabolites of photoautotrophic *Chlamydomonas reinhardtii* cultures grown under constant low/sub‐saturating (LL) or high/saturating (HL) light with 2% (CO_2_) or ambient 0.04% (Amb) CO_2_. HL‐Amb cells suffered photoinhibition and limitation of photosystem I electron flow at the donor side, but not the acceptor side, indicating use of alternative electron pathways to fuel ATP synthesis. Further, more glycolate was excreted under HL‐Amb, indicative of photorespiration. In contrast, HL‐CO_2_ cells upregulated the cytochrome *b*_6_ *f* complex, ascorbate metabolism, and PTOX2 for maintaining plastid redox homeostasis. Enhanced glycerol excretion under HL enabled dissipation of excess reducing equivalents to adjust the cellular energy balance. CO_2_‐enhanced photosynthesis promoted respiration and primary metabolite accumulation, driving faster growth while promoting nitrogen (N) metabolism. Hence, C_i_‐dependent photoacclimation influenced the interplay between the TCA cycle and N assimilation, as supported by proteomic data. Overall, abundant C_i_ supported growth by promoting electron flow for C_i_ assimilation, which supplied C skeletons for N assimilation while mitigating photorespiration and photoinhibition.

## 1 Introduction

Virtually all organic material on our planet is derived from photosynthesis, a process that provides the energy to all major ecosystems. Photosynthesis starts with light absorption by pigments—chlorophylls and carotenoids—in the light‐harvesting antenna, which transfer energy to initiate photochemistry in photosystem reaction centers. Photosystem II (PSII) uses this energy to split H_2_O, releasing electrons and protons, the latter of which accumulate in the thylakoid lumen. In the linear electron flow (LEF) of the so‐called electron transfer/transport chain (ETC), electrons are transferred from PSII towards photosystem I (PSI) via plastoquinol, the cytochrome *b*_6_ *f* complex (Cyt *b*_6_ *f*)—which also powers the transfer of protons to the lumen—and plastocyanin. Another light‐dependent step at PSI enables the reduction of the terminal electron acceptor, NADP^+^ for temporary storage of reducing power as NADPH, whereas the proton difference across the thylakoid membrane powers ATP production. NADPH and ATP are used to produce sugars in the Calvin–Benson–Basham (CBB) cycle, although LEF alone is not sufficient to match the required ATP:NADPH ratio (Johnson and Alric 2013). Moreover, other metabolic pathways require different ratios of ATP:NADPH; for example, under low inorganic carbon availability (C_i_), the operation of an ATP‐consuming carbon concentrating mechanism (CCM; Mackinder et al. 2017) is required to maintain efficient CO_2_ fixation. To meet varying energy demands, some reducing power needs either to be transported to the mitochondria to fuel ATP production via respiration or to be dissipated via alternative electron flows (AEFs). These include recycling reducing power back into the ETC via cyclic electron flow (CEF) upstream or into the Cyt *b*_6_ *f* complex, thereby promoting proton transfer to the thylakoid lumen and ATP synthesis at the cost of NADP^+^ reduction (Munekaga et al. 2004). In the chloroplast, AEFs also include the plastid terminal oxidase (PTOX) and flavodiiron proteins (FLV) that influence the redox poise of the ETC at separate locations: the plastoquinone pool via PTOX, and PSI via FLV. Furthermore, lumen acidification controls plastoquinol oxidation by Cyt *b*_6_ *f* and slows electron transfer towards PSI in so‐called photosynthetic control. In parallel to PTOX, the mitochondria also possess AEF via alternative oxidases (AOX) that oxidize the ubiquinone pool (Peltier et al. 2010; Eckardt et al. 2024). In summary, optimizing the cellular energy balance requires modulation of the ETC via AEF to match the variation in inputs (e.g., light and CO_2_ availability) to the required outputs (metabolites, reserves, cell division), but the relative contribution of each AEF pathway under optimal and suboptimal conditions is unclear (Burlacot 2023).

In the field, the chlorophyte alga *Chlamydomonas reinhardtii* thrives in temperate soil habitats, for example, muddy puddles out of direct sunlight, and thus is low‐light (LL) adapted. It can sense light via an eyespot, and its first response to excess light is flagella‐assisted negative phototaxis (Kreimer 2009). Excess light, hereafter referred to as high light (HL), describes light intensities that exceed the saturating threshold for C_i_ assimilation. When HL exposure is unavoidable, the production of reactive oxygen species (ROS), for example, hydrogen peroxide (H_2_O_2_) and singlet oxygen (^1^O_2_) increases, which contributes to photooxidative stress, including lipid peroxidation of thylakoid membranes (Roach et al. 2017). To prevent excess ROS production, short‐term responses to HL (min to hours) comprise enhanced AEF and the induction of photoprotective mechanisms, termed non‐photochemical quenching (NPQ; Erickson et al. 2015). A major component of NPQ is high‐energy state quenching (qE) that thermally dissipates excess energy, preventing photooxidative stress under HL (Roach et al. 2020). Furthermore, the xanthophylls, violaxanthin (V), antheraxanthin (A), and zeaxanthin (Z) inter‐convert in the VAZ cycle. Conversion from V to A to Z occurs under HL that protects the thylakoid membrane from lipid peroxidation (Niyogi et al. 1997; Baroli et al. 2003), with Z converting back to V under LL. Long‐term HL acclimation (hours to days) typically involves reduction in LHC antenna size, accompanied by changes in primary pigment composition to lower excitation pressure and prevent photooxidative stress, and/or changes of the PSI:PSII ratio to re‐tune the redox poise of the ETC and adjust the ATP:NADPH ratio (Humby and Durnford 2006; Erickson et al. 2015). When photodamage to PSII exceeds the repair capacity of the D1 protein (PsbA), PSII reaction centers are photoinactivated, which on the one hand lowers photosynthetic efficiency (photoinhibition), while on the other lowers undesired PSII activity and resulting photooxidative stress (Kok 1956). In microalgae, acclimation strategies are diverse and can vary even between species. For instance, *Dunaliella salina* decreases LHCs of both PS under HL (Smith et al. 1990), while *D. tertiolecta* changes the PSI:PSII ratio rather than antenna size (Falkowski and Owens 1980). In *C. reinhardtii* , HL induces a reduction in antenna size (Durnford et al. 2003; Pfleger et al. 2025), especially under elevated CO_2_ conditions (Polukhina et al. 2016), reflecting both conserved and divergent strategies among microalgae for acclimating to similar environmental conditions.

Photosynthetic organisms achieve only a fraction of the maximum theoretical solar energy conversion into glucose, estimated to 11% efficiency. Indeed, since algal cultures face adverse conditions such as high O_2_ concentrations and self‐shading in very dense cultures, the photosynthetic efficiency can be further reduced to 5%–7% efficiency, which nonetheless is slightly higher than typically achieved by C_3_ (3.5%) and C_4_ (4.3%) crops (Blankenship et al. 2011). Rapid adjustments of the C and nitrogen (N) metabolism to the environment are essential for balanced cellular homeostasis (Huppe and Turpin 1994; Davis et al. 2013). Microalgae's capacity to adjust their photosynthetic efficiency to different environmental conditions contributes to their high metabolic flexibility, which is superior to land plants, and enables them to inhabit various terrestrial and aquatic environments (Masojídek et al. 2013). Light, C_i_ and N availability not only govern immediate metabolism, but also shape long‐term energy sinks in algae. For example, N deficiency promotes starch and lipid synthesis in the absence of protein synthesis (Johnson and Alric 2013), whereas under C_i_ restriction the induction of the CCM leads to the formation of a starch sheath around the pyrenoid where the C_i_ fixing enzyme, RuBisCO, is located (He et al. 2023). Low CO_2_ concentrations around RuBisCO increase the likelihood that it reacts with O_2_ rather than CO_2_, leading to photorespiration, an ATP‐consuming and CO_2_‐producing pathway. Previous studies showed that photorespiration may occur in the presence of CCM at low CO_2_ (Dao et al. 2025). Indeed, putatively associated proteins accumulated in *C. reinhardtii* under high and fluctuating light in CO_2_ concentrations (0.04%) that induced the CCM (Pfleger et al. 2025).

Selective pressure imposed by contrasting environmental conditions can drive long‐term adaptation over generations, shaping an organism's capacity for acclimation. This process underlies *C. reinhardtii* 's ability to thrive under varying light and CO_2_ conditions. However, the precise interplay between the regulation of the ETC and the metabolic response to conditions that impose growth limitations remains unclear. To investigate this, we first characterized photosynthesis, including primary pigments and protein levels of major complexes, in *C. reinhardtii* acclimated to constant growth conditions that impose different energetic constraints, specifically sub‐saturating LL and saturating HL in the presence of ambient (Amb, 0.04%) and elevated (2%) CO_2_. We further conducted metabolite profiling of these cells to be able to relate differences in bioenergetics to the metabolic status, and quantified protein levels of PTOX2 and AOX1 for insights into the role of these AEF. The results were then integrated with our knowledge on various energy trade‐offs to provide an overall deeper insight into what governed cell metabolism and growth under each condition.

## 2 Material and Methods

### 2.1 Cultivation Conditions and Growth Assessment

The *C. reinhardtii* wild‐type (WT) strain CC‐4533 was kept at 25°C, on 1.5% agar supplemented with tris‐acetate‐phosphate (TAP) medium in a growth chamber (25 photons μmol m^−2^ s^−1^). Prior to the experiments, the cells were pre‐cultured using photoautotrophic Kropat's medium (Kropat et al. 2011) buffered with HEPES (adjusted to a pH of 7.5 with potassium hydroxide), in 1 L Schott bottles, incubated in a growth chamber at 25°C with continuous shaking (100 rpm) under continuous white light (100 μmol photons m^−2^ s^−1^) and bubbled with sterile pre‐humidified air. Upon reaching early exponential growth, the experimental cultures were transferred to 1 L flat‐panel photobioreactors FMT150 (PSI) and aerated with constant sterile and humidified air bubbling at an airflow of 0.5 L min^−1^ ambient (0.04% CO_2_, 21% O_2_) or elevated CO_2_ (2% CO_2_, 21% O_2_) and incubated at 25°C under either continuous HL (500 μmol photons m^−2^ s^−1^) or LL (50 μmol photons m^−2^ s^−1^) for at least 48 h. Growth was monitored by measuring the optical density at 720 nm (OD_720_) every 5 min with the photobioreactor sensors. A linear regression based on the dry weights of cells, sampled under the different culture conditions and freeze‐dried, against the cultures OD_720_ was used to convert the OD_720_ to an estimated dry biomass. To minimize self‐shading, all experiments were performed with cultures from two independent cultivation rounds, early in exponential growth at an OD_720_ of up to 0.3 that corresponded to a total chlorophyll concentration of ca. 2.5 μg mL^−1^.

### 2.2 High Resolution O_2_ Measurements

Net O_2_ production and consumption were measured using the PhotoBiology‐Module of a NextGen‐O2k (Oroboros Instruments), consisting of two 2 mL chambers each connected to a Clark‐type electrode (POS, polarographic O_2_ sensor). All experiments were conducted under constant stirring (750 rpm) at 25°C, and data points were collected at a frequency of 2 s. As the O_2_ chamber was a closed system during measurements, measurements were made in 5 mM NaHCO_3_ to ensure photosynthesis was saturated with CO_2_. Instrument calibration was performed with cell‐free medium according to Gnaiger (2001, 2008). The volume specific O_2_ flux (O_2_ flux per volume) was calculated as the time derivative of the O_2_ concentration corrected for instrumental background using the DatLab software (v8.0.3, Oroboros Instruments). Two milliliter of culture with similar OD_720_ was added to each chamber before the measurements. The reported O_2_ fluxes correspond to the median of 5–30 data points after stabilization of the signal, which were normalized to the dry biomass of the cells. O_2_ measurements were conducted at the light intensities under which the cells were grown. The peak of O_2_ consumption that occurred 30 s after switching off the light was used as a measure of light‐enhanced dark respiration (LEDR; Figure S1A). Gross O_2_ production was estimated by summing net O_2_ production and O_2_ consumption of LEDR. However, considering that LEDR does not fully account for the O_2_ consumption in the light, as several O_2_ consuming processes other than mitochondrial respiration (e.g., FLV, Mehler reaction) do not occur in the dark, gross O_2_ production may be underestimated.

### 2.3 Chlorophyll Fluorescence and P700 Measurements

For transient near‐infrared absorption of PSI reaction center chlorophyll (P700) and chlorophyll fluorescence measurements, 3 mL of culture was transferred to a cellulose acetate filter with 5 μm pore size (Sartorius) using a vacuum pump and a Buchner funnel. The filter and cells were placed between a transparent plastic films for immediate measurements using the leaf clamps of a DUAL PAM (Walz). Cells were first exposed for 3 min to 50 μmol photons m^−2^ s^−1^ and then for 3 min to 500 μmol photons m^−2^ s^−1^. At the end of each 3 min, an 800 ms saturating light pulse of 7044 μmol photons m^−2^ s^−1^ was given to calculate electron transport rates (ETR) of PSII and PSI, according to the equations provided in Schreiber et al. (1986) and Klughammer and Schreiber (1994). To prevent the deactivation of the CBB, cells were not dark‐treated. ETR values have not been adjusted to PSI or PSII levels.

### 2.4 GC–MS‐Based Metabolite Profiling

For metabolite profiling, cultures were grown to an OD_720_ of 0.3 ± 0.02 before harvesting. Cells and culture medium were harvested after at least 48 h of acclimation to the different growth conditions. In total, five samples were taken from each growth condition, comprising the replicates (*n* = 5). Metabolite profiling analysis was conducted identically to Pfleger et al. (2025) from separate cultures. Metabolite relative abundances were normalized to the dry biomass.

### 2.5 Pigment Analysis Using HPLC

For pigment quantification, 20 μL of the lipophilic phase collected during the GC–MS metabolite profiling extraction was transferred to dark HPLC vials. After evaporation under a stream of N_2_ for 10 min, pigments were resuspended in 0.5 mL 100% methanol, centrifuged at 26,000*g* for 45 min at 4°C, and 10 μL of supernatant were injected using an Agilent 1100 HPLC system equipped with a LiChrospher 100 RP‐18 (125 × 4 mm, 5 μm) column (Agilent Technologies). Identification and quantification were made using external standards of chlorophyll *a*, *b*, and β‐carotene (Sigma‐Aldrich), lutein, zeaxanthin (Carl Roth) and antheraxanthin, neoxanthin, and violaxanthin (DHI LAB Products). We were not able to distinguish loroxanthin and neoxanthin based on their elution; data refers to both compounds together. Pigment contents were normalized to the dry biomass.

### 2.6 Western‐Blotting

Proteins extracted from pellets collected during the GC–MS metabolite profiling extraction, as previously described (Pfleger et al. 2025), were used for LHCII and Cyt *f* detection. Alternatively, proteins were extracted from cell pellets collected after quenching by the addition of 60% methanol (1:1, v:v) pre‐cooled to −50°C and centrifugation (2000*g*, 3 min, −20°C) in 2% SDS in 50 mM TRIS at pH 6.8 with protease inhibitors (PsbA, PsbA, PTOX2, AOX1, CAT, and APX1). Proteins were denatured in 0.1 M DTT at 85°C for 5 min and separated by 1D‐SDS‐PAGE on 12% acrylamide gels, as previously described (Roach et al. 2020). Blotting was performed using nitrocellulose membranes for all antibodies except PsaA and PsbA, for which PVDF membranes were used, and proteins were detected using primary antibodies (from Agrisera, unless otherwise specified) for LHCII (against Lhcb2 in *Arabidopsis thaliana* ; AS01‐003), AOX1 (Cre09.g395950, AS06‐152), Cyt *f* (CreCp.g802263, AS06‐119), catalase (Cre09.g417150, AS15‐2991), APX1 (Cre02.g087700, AS15‐2992), at 1:10,000 dilution, against PTOX2 (Cre03.g172500, gift from Fabrice Rappaport) at 1:5000 dilution, and against PsbA antibody (CreCp.g802285, CreCp.g802321, AS05‐084) and PsaA (CreCp.g802280, CreCp.g802281, CreCp.g802282, AS06‐172) at 1:50,000 dilution, and detected using HRP conjugated Goat anti‐Rabbit IgG secondary antibody at 1:25,000 dilution (Agrisera) with the Amersham ECL Select western blotting detection reagent (Cytiva). Protein loading for Western Blot was adjusted based on the cultures OD_720_ and for each antibody. Relative quantification from gels and blots was performed using the ImageQuantTL software (v8.2, GE HealthCare) and values are expressed on a dry biomass basis.

### 2.7 Statistical Analysis

All statistical analyses were performed using R (v4.4.1, R Core Team 2024). For all univariate datasets, pairwise comparisons of the estimated marginal means were conducted using the “emmeans” package, on log_2_ transformed data after fitting a linear model, and *p* values were adjusted for multiple comparisons using the false discovery rate (FDR) correction. Proteomic data, previously generated for the exact same light and CO_2_ conditions (Pfleger et al. 2025), were re‐analyzed after excluding the fluctuating light treatments. Differential protein and metabolite accumulations were assessed after log_2_ transformation, using the Limma package (Ritchie et al. 2015) with a threshold set to 0.05 after global *p* value adjustment for multiple comparisons using the FDR correction. Heatmaps were generated using the ComplexHeatmap package (Gu et al. 2016) and additional figures using the ggplot2 package (Wickham 2016).

## 3 Results

### 3.1 Electron Fluxes and Growth Are Influenced by CO_2_ and Light Availability

**FIGURE 1:**
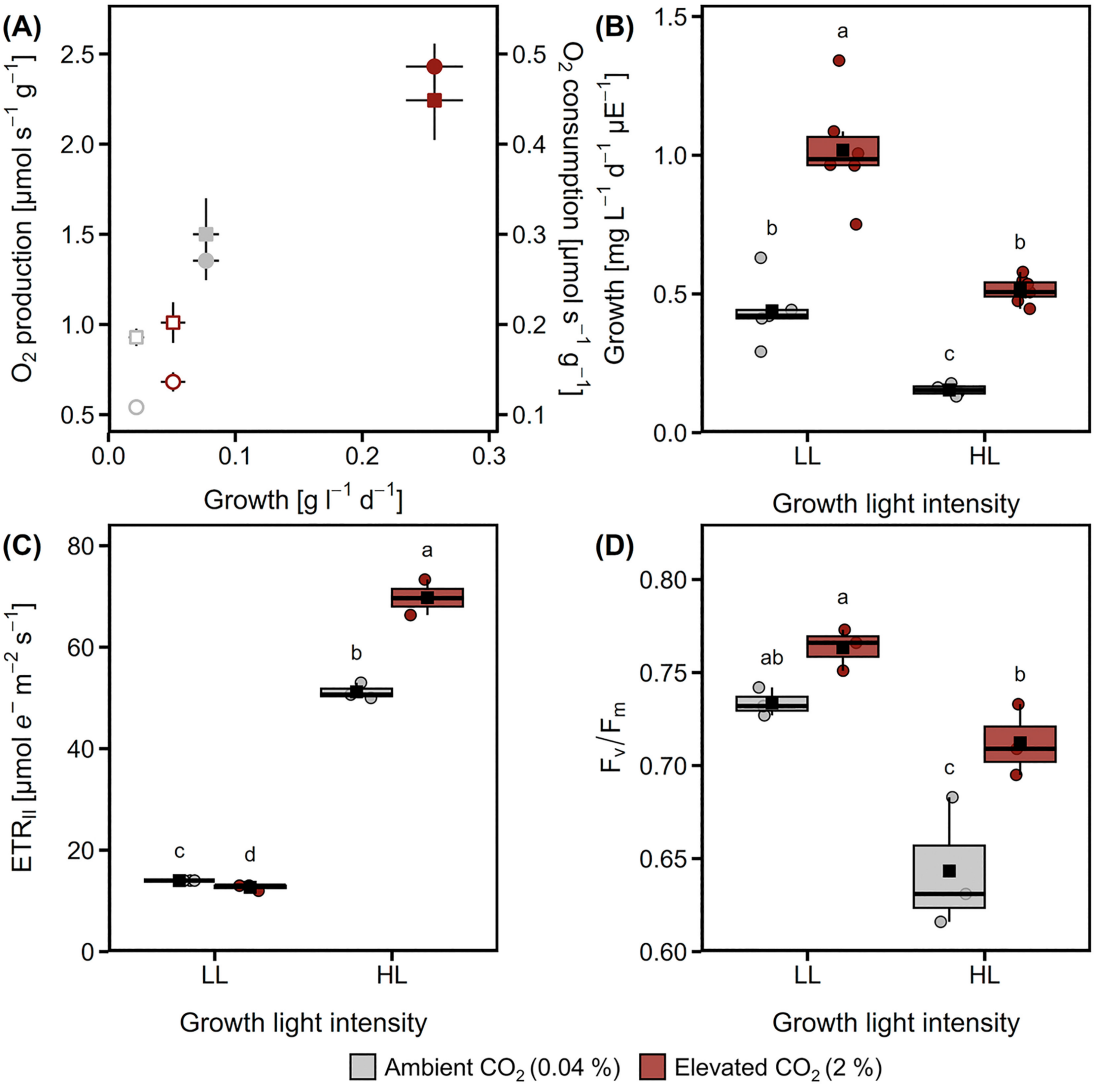
Influence of CO_2_ availability and light intensity on photosystem II (PSII) activity, respiration and culture growth rates. Cells were grown under low light (LL, 50 μmol m^−2^ s^−1^, open symbols in A) or high light (HL, 500 μmol m^−2^ s^−1^, closed symbols in A), with 0.04% (grey) or 2% CO_2_ (red). (A) Relationship between growth and gross O_2_ production, as a marker for PSII activity (circles; left *y* axis) and between growth and maximum O_2_ consumption immediately after switching off the light (squares; right *y* axis), as a marker for light‐enhanced dark respiration, under each culture condition. (B) Culture growth rates, as shown in (A), relative to light availability in each culture condition in μmol photons m^−2^ s^−1^ (μE). (C) Electron transfer rates of PSII (ETR_II_) and (D) maximum efficiency of PSII (*F*_v_/*F*_m_) after 30 min dark treatment, for each culture condition. Box plots show medians and the 25th and 75th percentiles, dots represent individual data points and black squares correspond to the means (*n* = 3). Different letters denote significant differences (FDR adjusted *p* < 0.05).

**FIGURE 2:**
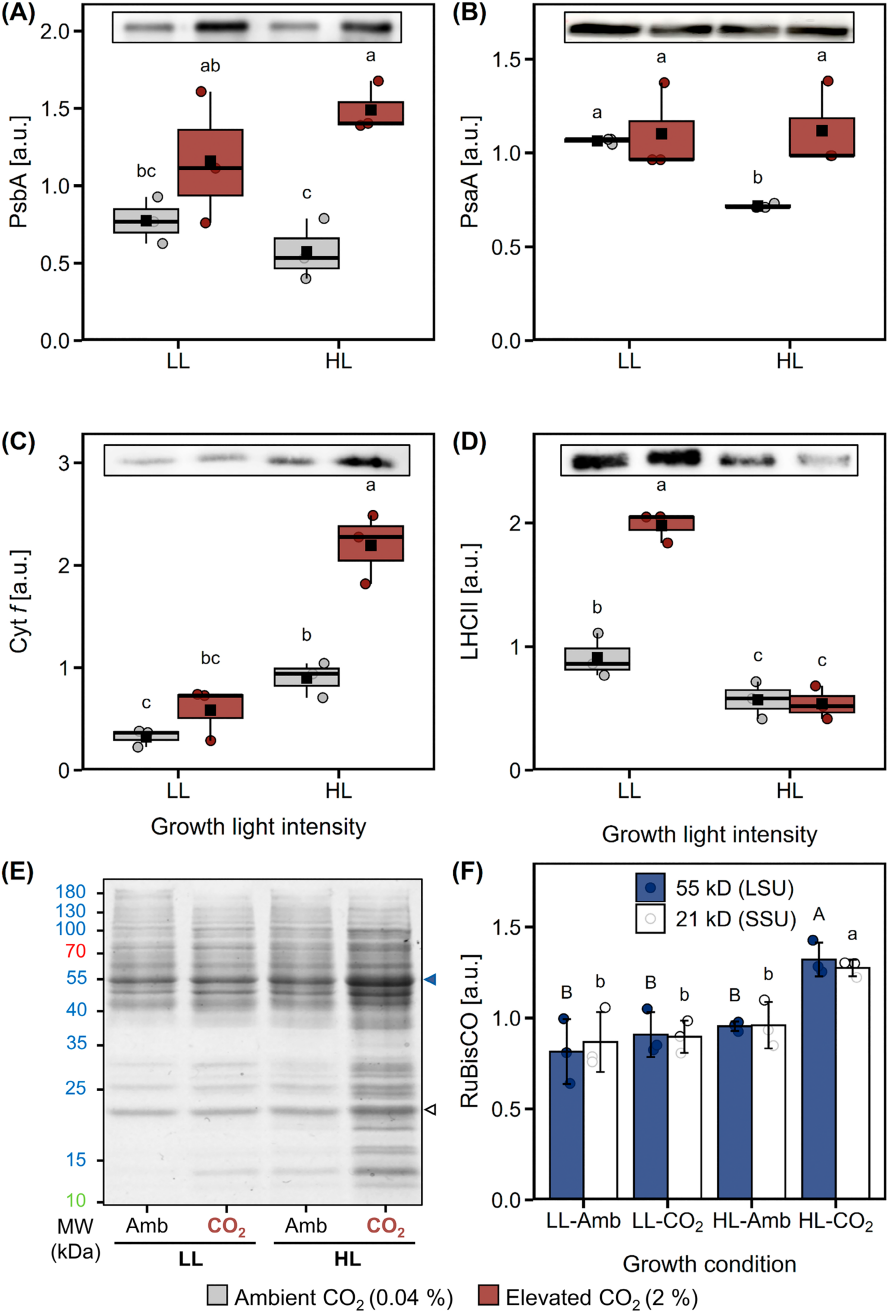
Influence of CO_2_ availability and light intensity on the accumulation of proteins involved in the photosynthetic electron transfer chain or carbon assimilation. Cells were grown under low light (LL, 50 μmol m^−2^ s^−1^) or high light (HL, 500 μmol m^−2^ s^−1^), with 0.04% (grey) or 2% CO_2_ (red). (A–D) Relative quantification of the signal intensity of the bands detected by western blot using antibodies against the photosystem II (PSII) subunit PsbA (A), the PSI subunit PsaA (B), the cytochrome *b*_*6*_ *f* complex subunit Cyt *f* (C) and light‐harvesting complex II (LHCII) (D). The insert above each plot displays the bands on a representative blot. Box plots show medians and the 25th and 75th percentiles, dots represent individual data points and black squares correspond to the means (*n* = 3). All densitometry quantification is relative to dry biomass. (E) Representative 1D‐SDS‐PAGE gel obtained from total protein extracts and stained with Coomassie blue, indicating the position of the bands corresponding to the large (LSU, blue arrow) and small (SSU, white arrow) subunits of the ribulose‐1,5‐bisphosphate carboxylase/oxygenase (RuBisCO). (F) Relative quantification of the signal intensity associated to LSU and SSU after Coomassie staining. Different letters denote significant differences (FDR adjusted *p* < 0.05; on panel (F), small and capital letters, respectively, correspond to the small and large RuBisCO subunits).

HL and CO_2_ had an interactive effect in increasing the growth of *C. reinhardtii* in photoautotrophic cultures, as reflected by the slowest increase in culture biomass observed for cells grown under LL with ambient CO_2_ (LL‐Amb), and fastest for cells grown under HL with 2% CO_2_ (HL‐CO_2_) (Figure 1A). Rates of gross O_2_ production, as a putative marker of PSII activity (i.e., photosynthesis), and of maximum light‐enhanced O_2_ consumption in the dark (i.e., respiration), both positively correlated with culture growth rates (Figure 1A; Figure S1A). However, the ratio of O_2_ production to O_2_ consumption was not equal across all growth conditions, but increased with the growth rate from 2.9 for LL‐Amb cells to 5.4 for HL‐CO_2_ cells (Figure S1). When growth rates were adjusted for photons available, LL‐CO_2_ cultures had the highest light‐use efficiency for biomass production and HL‐Amb the lowest (Figure 1B). These differences were reflected in the maximum efficiency of PSII (*F*_v_/*F*_m_; Figure 1D) and in the electron transport rates (ETR) of PSII (ETR_II_) for HL cultures (Figure 1C) that grew the fastest (Figure 1A). Levels of PsbA, the D1 reaction center of PSII, were lower in HL‐Amb compared to HL‐CO_2_ cultures (Figure 2A). Overall, these results showed that PSII levels and efficiency were both fundamental to growth and influenced by C_i_ and light availability.

**FIGURE 3:**
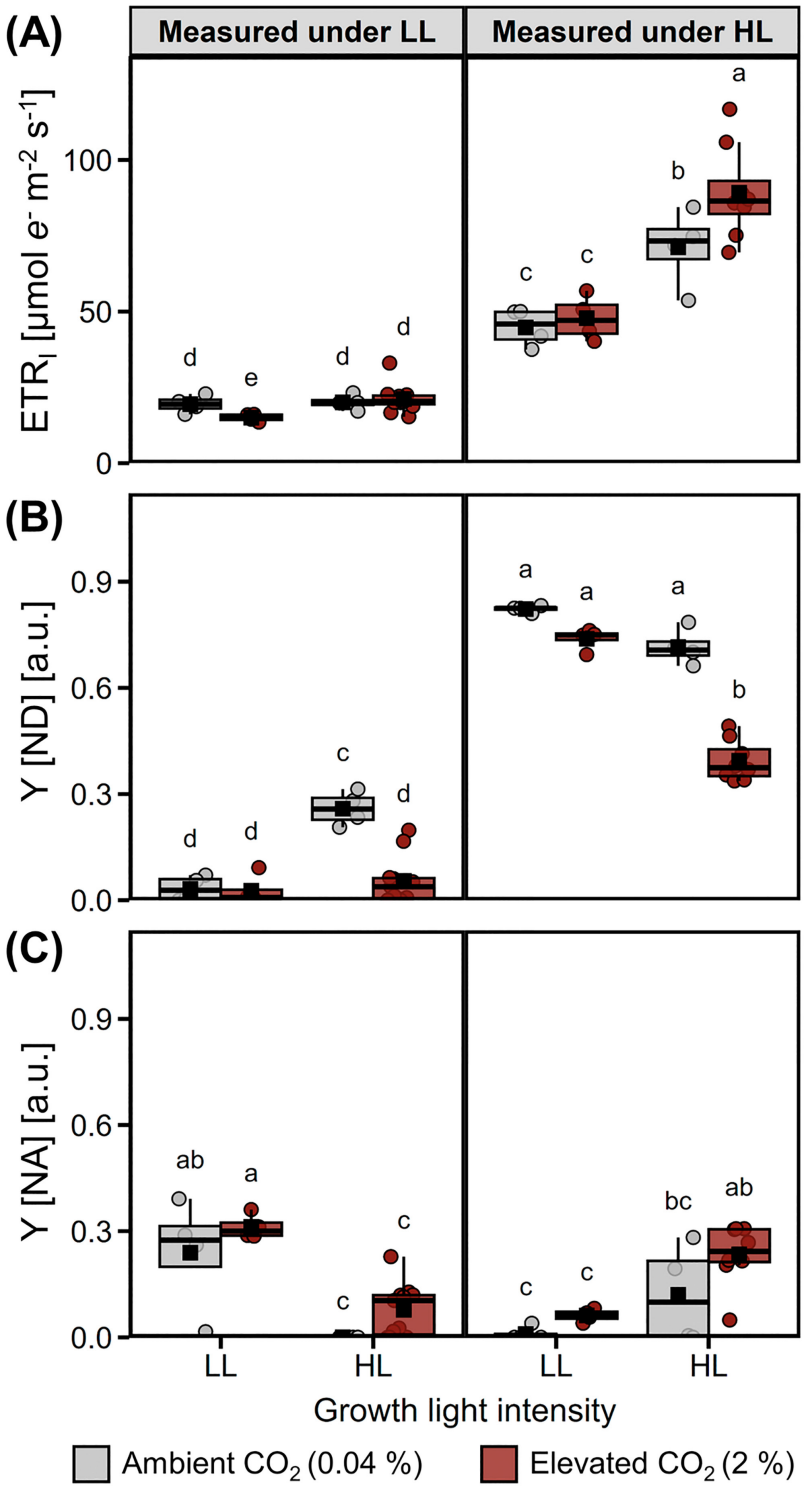
Influence of CO_2_ availability and light intensity on photosystem I (PSI) activity. Cells were grown under low light (LL, 50 μmol m^−2^ s^−1^) or high light (HL, 500 μmol m^−2^ s^−1^), with 0.04% (grey) or 2% CO_2_ (red), and PSI activity was measured under LL (left panels) or HL (right panels). (A) Electron transfer rates of PSI (ETR_I_) and limitations to ETR_I_ due to (B) limited PSI electron donors (Y[ND]), and (C) limited PSI electron acceptors (Y[NA]), on a scale between 0 (no limitation) and 1 (fully limited). Absorption changes of PSI used for calculating values are available in Figure S2. ETR_I_ are not normalized to PSI content. Box plots show medians and the 25th and 75th percentiles, dots represent individual data points and black squares correspond to the means (*n* = 3–8). Different letters denote significant differences (FDR adjusted *p* < 0.05).

ETR of PSI (ETR_I_) revealed that acclimation to HL enhanced PSI electron flow capacity, with HL‐grown cells exhibiting higher ETR_I_ than LL‐grown cells when measured under HL (Figure 3A). This occurred despite lower levels of PsaA, the PSI‐A core reaction center, in HL‐Amb compared to LL‐Amb cells (Figure 2B), implying that factors aside from PSI levels limited ETR_I_. These include donor side (Y[ND]) and acceptor side (Y[NA]) limitations on PSI electron flow, whereby under HL, Y[ND] dominated and HL‐Amb cells had more Y[ND] than HL‐CO_2_ cells (Figure 3B). Instead, under LL, LL cells had more Y[NA] whereas HL cells had virtually none (Figure 3C), indicating that HL acclimation increased PSI electron acceptor capacity. Overall, acclimation to 2% CO_2_ tended to decrease Y[ND], but had hardly any impact on Y[NA], indicating that electron flow towards PSI was more affected by CO_2_ availability than electron flow away from PSI. A major factor influencing Y[ND] is the rate of electron flow through Cyt *b*_6_ *f*, and levels of Cyt *f* were lowest in LL cells and highest in HL‐CO_2_ cells (Figure 2C).

### 3.2 Acclimation to CO_2_ and Light Availability Involved Adjustments in Photosynthetic Pigments

**FIGURE 4:**
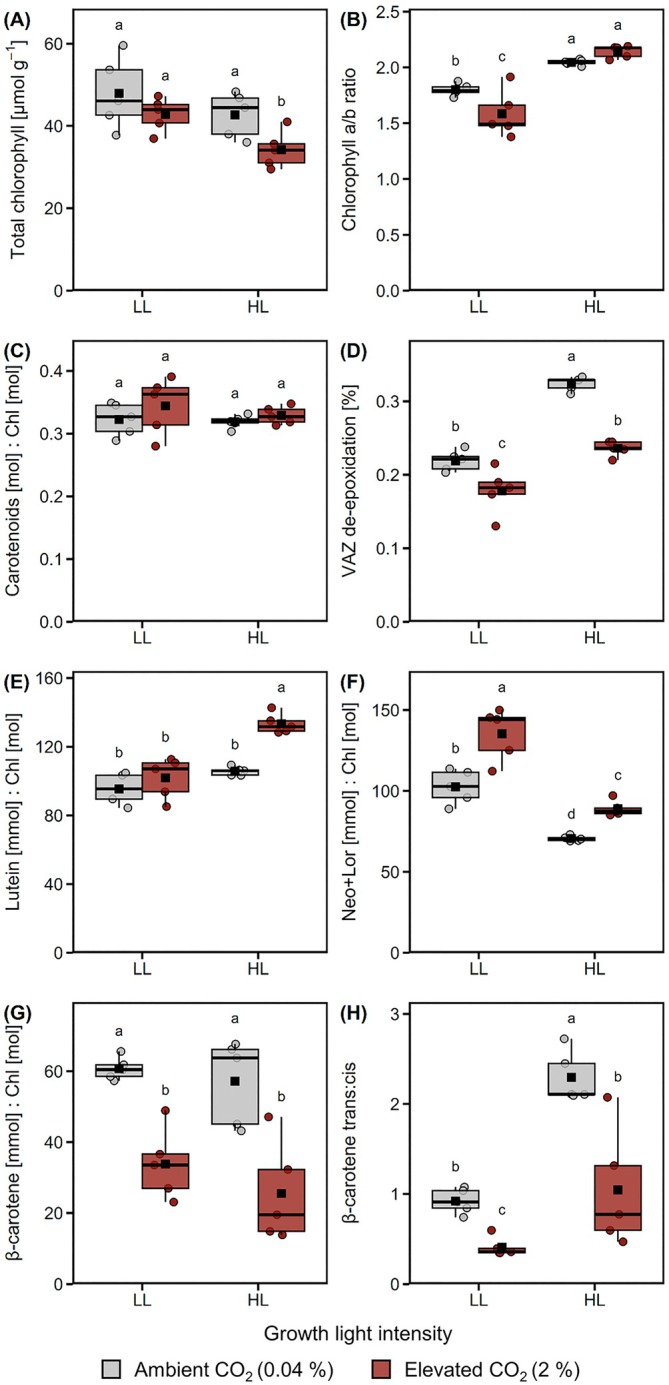
Influence of CO_2_ availability and light intensity on the accumulation of primary pigments. Cells were grown under low light (LL, 50 μmol m^−2^ s^−1^) or high light (HL, 500 μmol m^−2^ s^−1^), with 0.04% (black) or 2% CO_2_ (red). (A) Total chlorophyll (Chl) concentration of the culture was normalized to dry weight. (B) Mol ratio of Chl *a*/*b*. (C) Total carotenoids relative to total Chl. (D) De‐epoxidation ratio of violaxanthin (V), antheraxanthin (A) and zeaxanthin (Z) calculated by (A + Z)/(V + A + Z). Amounts of (E) lutein, (F) neoxanthin and loroxanthin (Neo + Lor), and (G) β‐carotene, relative to total Chl. (H) Ratio of *trans*:*cis* isomers of total β‐carotene. Box plots show medians and the 25th and 75th percentiles, dots represent individual data points and black squares correspond to the means (*n* = 5). Different letters denote significant differences (FDR adjusted *p* < 0.05).

Total chlorophyll concentrations were lower under HL‐CO_2_ than under any other condition (Figure 4A), while the chlorophyll *a*:*b* ratios were higher under HL (2.0–2.1) than under LL (1.4–1.7), and highest in HL‐CO_2_ cells (Figure 4B). The PSII light‐harvesting antennae contain the majority of chlorophyll *b*, and in agreement with differences in chlorophyll *a*:*b* ratios, LHCII levels were also lower in HL than in LL cells and highest in LL‐CO_2_ cells (Figure 2D). Total carotenoid concentrations relative to total chlorophyll did not change across growth conditions (Figure 4C), but the carotenoids composition did. The de‐epoxidation ratio of the VAZ cycle was higher under HL than LL, even more so in Amb as compared to CO_2_ cells (Figure 4D). Amounts of lutein were highest in HL‐CO_2_ cells (Figure 4E), while neoxanthin/loroxanthin amounts were higher in LL cells, as well as in CO_2_ than in Amb cells under HL and LL (Figure 4F). In contrast, amounts of β‐carotene were higher in Amb than in CO_2_ cells (Figure 4G). The photosystem antennas contain all‐*trans* β‐carotene, whereas *cis* isomers have been found in PSII and Cyt *b*_6_ *f*, specifically 15‐*cis* and 9‐*cis* isomers, respectively (Bialek‐Bylka et al. 1995; Yan et al. 2001). The 15‐*cis* isomer can isomerize to all‐*trans* β‐carotene during pigment extraction (Bialek‐Bylka et al. 1995), whereas the 9‐*cis* isomer is retained, with the β‐carotene absorption spectra detected here (Figure S3) typical of 9‐*cis* (Yan et al. 2001). The β‐carotene *trans*:*cis* ratio differed with light intensity, with LL cells having twice as much *cis* isoform as HL cells (Figures 4H and S3).

### 3.3 Elevated CO_2_ Availability Promotes RuBisCO Accumulation and Redox Regulation Under High Light

Coomassie‐stained protein band densities (i.e., amount) were overall highest in HL‐CO_2_ cells, particularly at regions of ca. 55 and 21 kD (Figure 2E,F), corresponding to the sizes of the large and small subunits of RuBisCO, respectively. Considering that RuBisCO is typically the most abundant protein in photosynthetically active cells and may represent more than 10% of the total proteins even under mixotrophic conditions (Hammel et al. 2018), this result presumably reflects higher RuBisCO levels under HL‐CO_2_.

In line with major changes in protein levels due to the growth conditions (Figure 2E), analysis of proteomic data generated from cells grown in the same bioreactors and under the same growth conditions as used here (Pfleger et al. 2025) revealed moderate changes in protein levels involved in ammonium and amino acid metabolism (Tables S1 and S2; Figure S4). The level of various oxidase enzymes, including the H_2_O_2_‐catabolizing APX1 and CAT, was also influenced by light and C_i_ availability. While both antioxidants were more abundant in cells grown under HL than under LL, plastid‐localized APX1 was particularly abundant in HL‐CO_2_ cells (Figure S5). The highest levels of PTOX2 were also found in HL‐CO_2_ cells, whereas AOX1 in the mitochondria was slightly higher in HL‐Amb cells (Figure S5).

In summary, high C_i_ availability played a key role in photosynthetic acclimation to HL, resulting in the most pronounced effect on culture growth rates. This included a prevention of loss of PSII and PSI reaction center numbers and in the decline in PSII efficiency, and increased levels of Cyt *f* and RuBisCO. However, in LL cells, the influence of high C_i_ availability was also apparent in the high level of PSII antenna, with LL‐CO_2_ cultures achieving the highest light use efficiency for growth.

### 3.4 Metabolite Accumulation Was Promoted by Increasing CO_2_ and Light Availability

**FIGURE 5:**
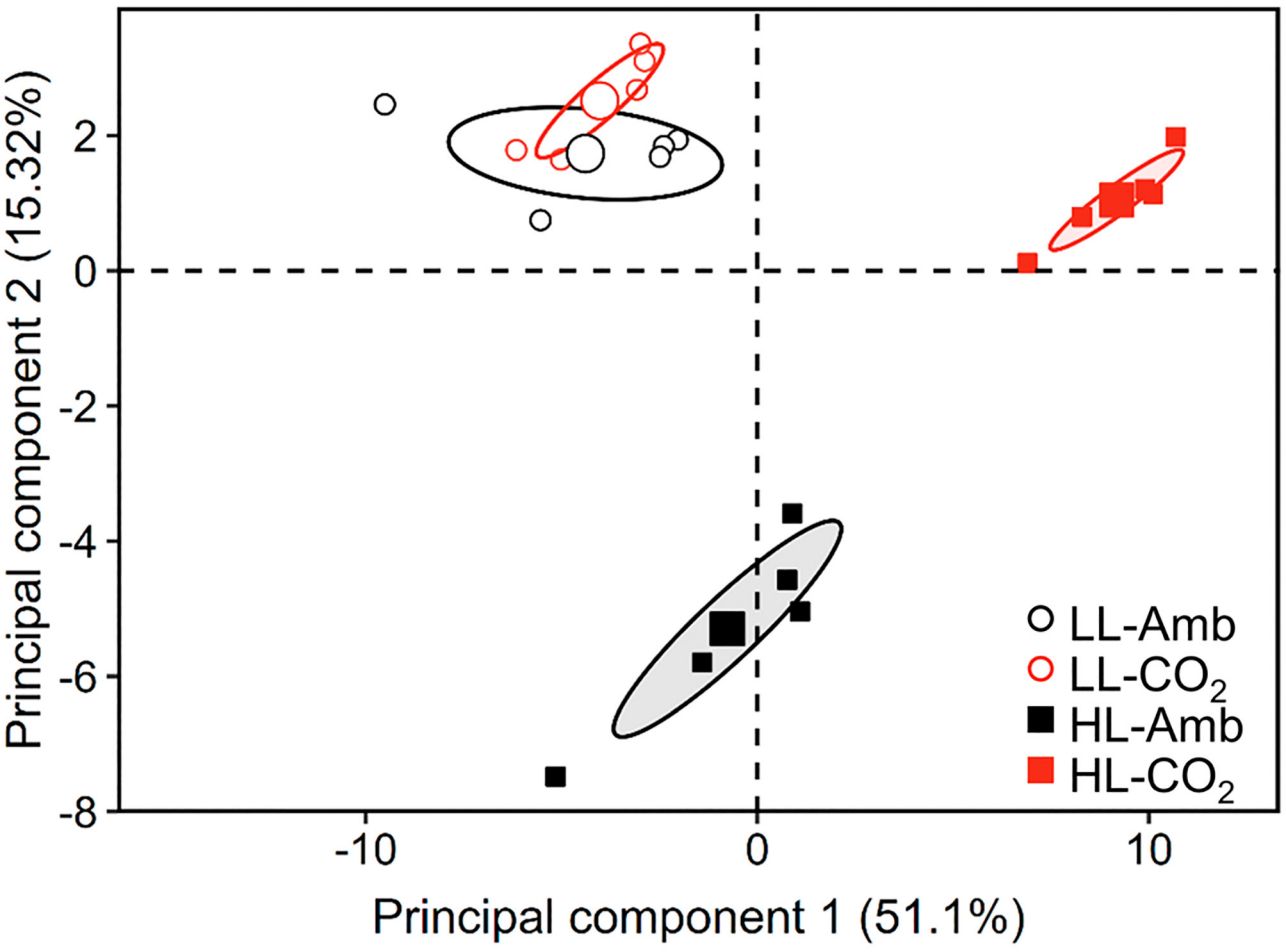
Overall influence of CO_2_ availability and light intensity on the metabolite profiles. Cells were grown under low light (LL, 50 μmol m^−2^ s^−1^, open circles) or high light (HL, 500 μmol m^−2^ s^−1^, filled squares), with 0.04% (black) or 2% CO_2_ (red). Samples were separated based on their metabolite profiles on the score plot of a principal component analysis, which was performed using the *Z*‐scores of log_2_ transformed metabolite relative abundances considering all compounds identified. The first two principal components, that is, linear combinations of the original variables selected to capture most of the variance in the dataset, are shown. Ellipses depict the 95% confidence intervals around the group means (larger symbols; *n* = 5).

**FIGURE 6:**
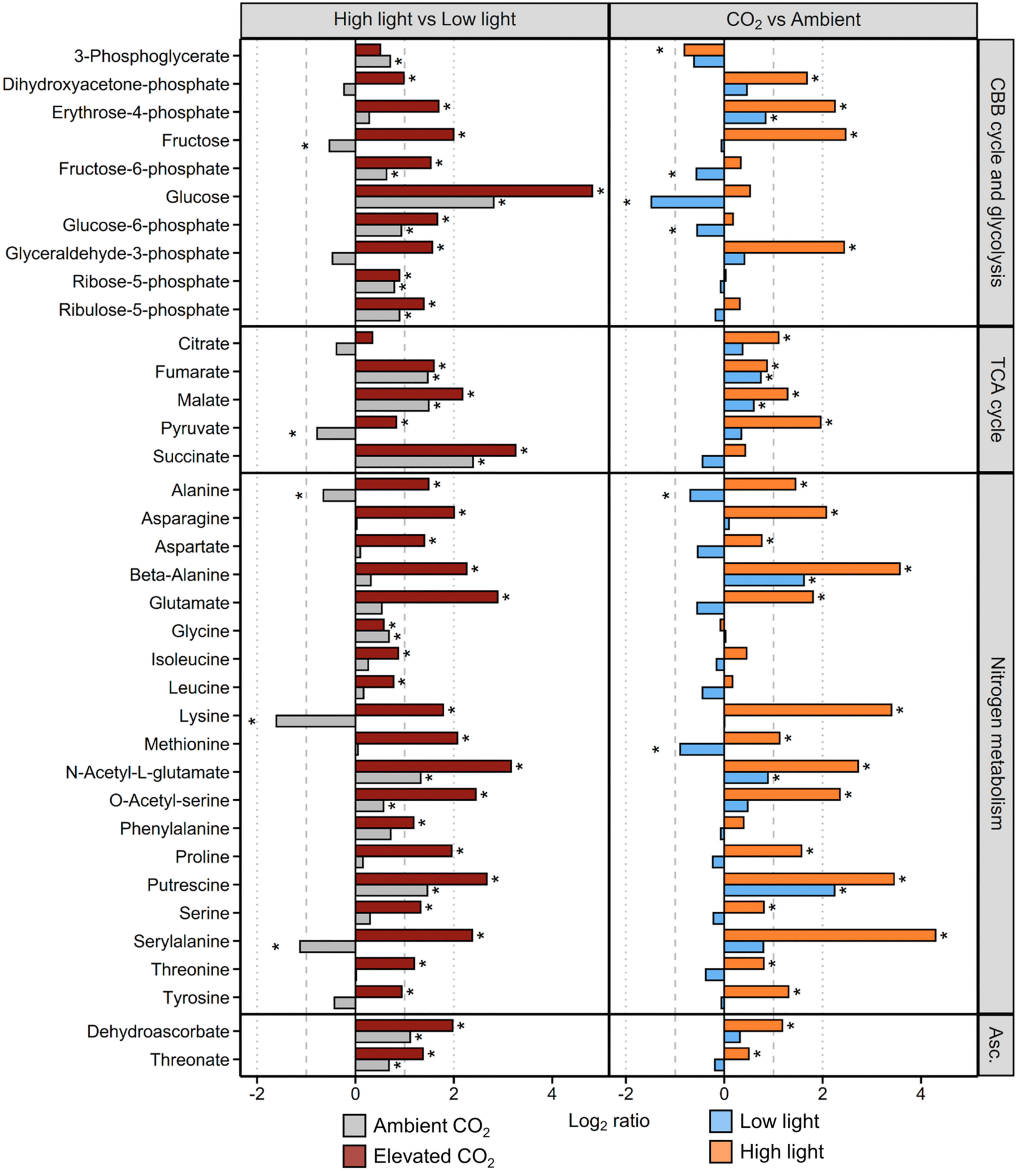
Influence of CO_2_ availability and light intensity on primary metabolite levels. Cells were grown under low light (LL, 50 μmol m^−2^ s^−1^) or high light (HL, 500 μmol m^−2^ s^−1^), with 0.04% (Amb) or 2% CO_2_ (CO_2_). Metabolites differential accumulation between HL and LL under either Amb (grey) or CO_2_ (dark red) is shown on the left, and between CO_2_ and Amb under LL (blue) or HL (orange) on the right. Data are log_2_ ratios of metabolite relative contents per dry biomass (HL/LL on the left, CO_2_/Amb on the right). Asterisks above the bar indicate statistical significance (FDR adjusted *p* < 0.05). Only selected primary metabolites including intermediates of the Calvin–Benson–Bassham (CBB) cycle, glycolysis and gluconeogenesis pathway, tricarboxylic acid (TCA) cycle, nitrogen and ascorbate (Asc.) metabolisms, are shown. Note that metabolites involved in the CBB cycle, glycolysis and gluconeogenesis were regrouped under “CBB cycle and glycolysis.”

A total of 72 metabolites were detected with GC–MS, out of which 66 showed significant variations between experimental conditions (Tables S3 and S4). Principal component analysis (PCA) showed that the primary metabolite profiles clearly differed depending on light and CO_2_ conditions as illustrated by the clustering along the first two components (PC) of the score plot, together accounting for 66.4% of the total variance. The most pronounced differences were observed under HL‐CO_2_ as evidenced by the separation along PC 1 (Figure 5). Light availability affected the accumulation of 61 metabolites (Figure S6), with HL mostly leading to higher contents than LL. Specifically, HL resulted in an increased accumulation for 55 metabolites under elevated CO_2_ and 27 under Amb, out of which 25 accumulated regardless of CO_2_ availability (Figure S6A). Indeed, many intermediates from the Calvin–Benson–Bassham (CBB) cycle, glycolysis/gluconeogenesis pathway, and tricarboxylic acid (TCA) cycle showed similar accumulation under HL as compared to LL in Amb and CO_2_ conditions, whereas glyceraldehyde‐3‐phosphate, dihydroxyacetone‐phosphate, pyruvate, and most amino acids only substantially accumulated under HL‐CO_2_ (Figure 6). The availability of C_i_ affected the accumulation of 50 metabolites. Specifically, CO_2_ promoted the accumulation of 32 metabolites exclusively under HL, two only under LL, and 10 under both light conditions (Figure S6C). Overall, the additive effect of HL and elevated CO_2_ resulted in the highest primary metabolite accumulation under HL‐CO_2_.

### 3.5 High Light Promoted Glycerol Excretion Whereas Elevated CO_2_ Led to Glycolate Release

**FIGURE 7:**
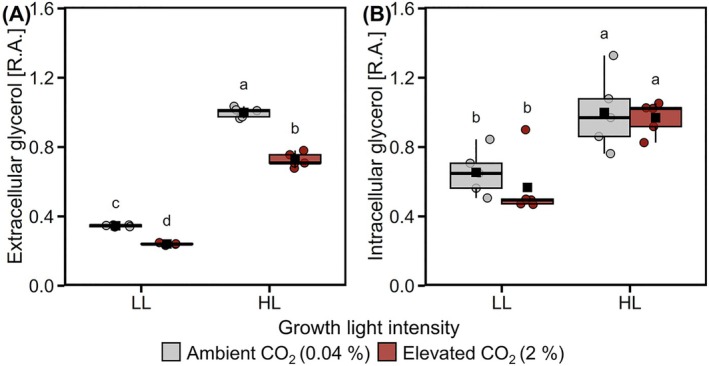
Influence of CO_2_ availability and light intensity on extra‐ and intra‐cellular glycerol accumulation. Cells were grown under low light (LL, 50 μmol m^−2^ s^−1^) or high light (HL, 500 μmol m^−2^ s^−1^), with 0.04% (grey) or 2% CO_2_ (dark red). Glycerol content was determined by GC–MS in the medium (A) and in the cells (B) and expressed as a relative abundance per dry biomass. Box plots show medians of relative abundance (R.A.) and the 25th and 75th percentiles, dots represent individual data points and black squares correspond to the means (*n* = 5). Different letters denote significant differences (FDR adjusted *p* < 0.05).

Glycerol was the main metabolite detected in the growth medium. Extra‐ and intracellular glycerol concentrations were significantly higher under HL than LL. CO_2_ availability did not substantially affect intracellular glycerol, but led to lower extracellular content under LL and HL (Figure 7). The ratio of total extra‐ to intracellular glycerol indicated that the extracellular concentration was much higher than within the cells, supporting that glycerol was excreted. Glycolate was only detected extracellularly and was at higher concentrations in the media of Amb compared to CO_2_ cells (Figure S7).

## 4 Discussion

The aims of this study were to improve the understanding of how cellular bioenergetics, including light‐harvesting, regulation of electron fluxes, and primary metabolism, adjust and acclimate in accordance with changing availability of CO_2_ and light that drive growth rates in *C. reinhardtii* .

### 4.1 PSII Drove Biomass Accumulation Under Photoautotrophic Conditions

Together, the strong positive correlation between rates of O_2_ production and culture growth (Figure 1A), as well as between light use efficiency for growth and *F*_v_/*F*_m_ (Figure 1B,D), shows the importance of PSII activity in cellular growth under a broad range of photoautotrophic growth conditions. The D1 reaction center of PSII, PsbA, has been described as the “engine of life” (Barber 2003) since it initiates photochemistry for C assimilation, which is the first step in the production of virtually all organic matter on our planet. However, PsbA has a very short half‐life due to oxidative damage incurred from the highly charged reactions involving the bound chlorophyll and electron transfer (Kale et al. 2017), leading to damaging ^1^O_2_ production (Krieger‐Liszkay 2005). Damage to PSII, known as photoinhibition, has energetic costs in terms of ATP, particularly due to necessary translation for renewing D1 (Quigg and Beardall 2003), and can be detected via decreases of *F*_v_/*F*_m_. As expected, due to more PSII damage under HL (Tyystjarvi and Aro 1996), *F*_v_/*F*_m_ values were lower for HL cells than for LL cells, and were the lowest for HL‐Amb cells (Figure 1D) that produce high levels of ^1^O_2_ (Roach et al. 2017). Previously, we showed that HL‐Amb cells had slightly faster rates of PSII damage than HL‐CO_2_ cells, although the lower *F*_v_/*F*_m_ in HL‐Amb cells was also explained by their very low ATP concentrations that would limit PsbA repair (Pfleger et al. 2025). Indeed, the lowest PsbA levels were found in HL‐Amb cells (Figure 2A). This highlights a major energetic trade‐off of photosynthesis; a significant fraction of the ATP/NADPH produced is consumed for maintaining PSII activity to enable ATP/NADPH production, at the expense of other metabolic processes that would promote growth.

### 4.2 CO_2_ Enhanced Electron Flux Through PSI and Modified Electron Acceptors

While antenna levels and NPQ alter photosystem activities, the redox poise of the ETC is also under regulation affecting ETR. ETR_I_ can be limited at the donor [to] and acceptor [from] sides of PSI. Y[NA] prevailed over Y[ND] in LL cells (Figure 3) and the higher Y[NA] of LL cells compared to HL cells when measured under LL (Figure 3C) showed that HL acclimation increased the availability of PSI electron acceptors, which likely contributed to higher ETR_I_ under HL (Figure 3A). The higher Y[ND] of HL‐Amb cells compared to HL‐CO_2_ cells, which increased under HL compared to LL (Figure 3B), can be explained by low luminal pH upregulating photosynthetic control that limits electron flow to PSI (Schreiber and Klughammer 2008). With 2% CO_2_, PSII was more abundant and had higher efficiency due to less photoinhibition and qE (Pfleger et al. 2025), resulting in higher electron flux to PSI. Excess reducing power can be diverted to PTOX2, which was more abundant under HL‐CO_2_ (Figure S5). In contrast, in Amb cells, AEFs from PSI have been shown to be upregulated (Burlacot 2023; Zuliani et al. 2024). Considering the low Y[NA] under HL and its lack of difference between HL‐CO_2_ and HL‐Amb, HL cells modified the electron paths downstream from PSI depending upon CO_2_ availability. In particular, FLV were more prevalent in HL‐Amb cells than in HL‐CO_2_ cells (Pfleger et al. 2025), indicating that this protein can act as a safety valve of the ETC when insufficient C_i_ is available for C assimilation. Although a waste of reducing power that otherwise could be used to fuel CEF and ATP production, FLV mitigates H_2_O_2_ production due to the HL induction of the non‐catalyzed O_2_ reduction pathway (i.e., Mehler reaction; Pfleger et al. 2024).

The observed accumulation of dehydroascorbate and threonate, two products of ascorbate oxidation, under HL (Figure 6) likely reflected an induction of the ascorbate metabolism in response to HL, as previously reported (Davis et al. 2013; Lin et al. 2016). The final step of ascorbate biosynthesis couples l‐galactono‐lactone oxidation to the reduction of Cyt *c* in the mitochondria (Wheeler et al. 1998). Therefore, the notably high respiratory rates in HL‐CO_2_ cells may also promote ascorbate synthesis. Moreover, higher APX1 levels in HL‐CO_2_ cells (Figure S5) and a higher dehydroascorbate reductase level in HL‐CO_2_ relative to HL‐Amb cells (Pfleger et al. 2025) agree with previous observations that high C_i_ availability increases rates of H_2_O_2_ production under HL (Roach et al. 2015). H_2_O_2_ production could derive from various sources, including the Mehler reaction from PSI, other components of the ETC (e.g., PSII), the mitochondrial ETC, and photorespiration. AOX1 was also slightly more abundant in HL‐Amb cells (Figure S5), potentially to prevent mitochondrial ROS production.

In summary, HL‐CO_2_ cells acclimated better to HL with lower Y[ND] to be able to use more photons for electron flow than HL‐Amb cells, while part of this acclimation also involved elevated redox control. Instead of primarily relying on photoprotective mechanisms leading to energy dissipation, high C_i_ availability promoted antioxidant metabolism to maintain redox homeostasis while maintaining high ETR. Despite that ATP is in short supply in HL‐Amb cells, CEF does not seem to represent a useful strategy to dissipate excess reducing power (Pfleger et al. 2025); potentially because the lumen is already at a low pH that limits electron flow by photosynthetic control, as interpreted by the high Y[ND].

### 4.3 Adjustments in Photosynthetic Pigments Reflected Distinct Photoacclimation Strategies Depending on CO_2_ Availability

To prevent photodamage under HL, changes occur at the level of the thylakoid membrane, including lowering chlorophyll content (Durnford et al. 2003; Bonente et al. 2012) and associated antenna pigments, such as neoxanthin, as observed here (Figure 4). Elevated C_i_ further promoted the decrease in total chlorophyll content, as previously described (Polukhina et al. 2016). The higher chlorophyll *a*:*b* ratio in HL cells (Figure 4B) agrees with less chlorophyll *b*‐rich PSII light‐harvesting antenna, for example, LHCII (Figure 2D), compared to LL cells. Another acclimation strategy photosynthetic organisms employ to prevent O_2_‐mediated photodamage is to dissipate excess absorbed light energy before it reaches the reaction center via LHCSR3‐mediated qE (Peers et al. 2009; Roach et al. 2020). Both qE and LHCSR3 levels were increased in HL‐Amb as compared to HL‐CO_2_ cells, showing a need for Amb cells in dissipating excess light energy (Pfleger et al. 2025). Carotenoids are also integral to photoprotection, and under HL, the de‐epoxidation of V to A and Z occurs, which may function in slowly forming qE and as an antioxidant (Niyogi et al. 1997; Baroli et al. 2003). The highest VAZ de‐epoxidation ratio of all conditions was found in HL‐Amb cells (Figure 4D), which needed a high level of photoprotection. Although lutein contributes to qE in LHCSR3 (Bonente et al. 2011; Zheng et al. 2024), it is also an integral antenna carotenoid, and the higher lutein levels in HL‐CO_2_ cells that have low qE could be part of the acclimation towards efficient light harvesting for these cells to achieve high ETR. A rate‐limiting step of ETR via LEF is the Cyt *b*_*6*_ *f* that contains 9‐*cis* β‐carotene (Bialek‐Bylka et al. 1995; Yan et al. 2001), detected here, with double the level in LL cells relative to HL cells (Figures 4H and S3). However, the lower levels of Cyt *f* (Figure 2C), along with higher 9‐*cis* β‐carotene levels in LL cells, indicated the reason for a higher *trans*:*cis* ratio in HL cells is due to other factors. For example, 9‐*cis* β‐carotene is found in the eyespot (Kreimer 2009). Potentially, LL cells have a larger eyespot due to light‐limited conditions, which needs further investigation. Overall, the pigment changes support that LL cells maximized light absorption (elevated chlorophyll and carotenoids), while C_i_ availability modulated pigment allocation to protection (VAZ; Amb) and light harvesting (lutein, neoxanthin; CO_2_) in HL cells.

### 4.4 Growth Rates Reflected Shifts in Cellular Energetics

Under photoautotrophic conditions, *C. reinhardtii* is strictly reliant on light to produce ATP and NADPH to power its metabolism for C assimilation, cell maintenance, and ultimately cell growth. Previously, we have shown, in cells grown under the exact same conditions as here, that CEF is upregulated under LL, along with changes in protein abundance presumably indicative of increased shuttling of organic acids (e.g., malate valve) to the mitochondria, likely to fuel the TCA cycle and respiration (Pfleger et al. 2025). Further support for this bioenergetic pathway was found in the lower gross O_2_ production in the light relative to LEDR in LL cells (Figure S1B), showing their greater reliance on respiration to produce ATP within the total cellular energetic balance. Acclimation to HL led to greater capacity for electron fluxes, including respiration, but also led to higher O_2_ production relative to LEDR, particularly under high C_i_ availability, indicating respiration made a relatively lesser contribution to total cellular ATP production than under LL.

### 4.5 Acclimation to Light and CO_2_ Availability Encompassed Adjustments of the Central C and N Metabolism

The onset of distinct acclimative strategies depending on light intensity and C_i_ availability may have a substantial impact on the energy available to sustain cellular metabolic activity. Growth under HL generally resulted in the accumulation of CBB cycle and TCA cycle intermediates (Figure 6) suggesting higher metabolic fluxes through the chloroplastic and mitochondrial energy pathways. Elevated CO_2_ further promoted the accumulation of primary metabolites, most prominently under HL, indicating that C_i_ availability became more restrictive to metabolism with increasing light intensity. Higher RuBisCO levels under HL‐CO_2_ (Figure 2F) presumably enhanced cells' C_i_ fixation capacity. The state of the cellular energy balance may substantially govern C allocation to biomass production, whereby HL‐CO_2_ was shown to promote both storage starch and protein accumulation (Pfleger et al. 2025). Instead, the induction of the CCM under Amb conditions, amplified under HL, includes the investment of C skeletons into the formation of a starch sheath around the pyrenoids (He et al. 2023). An induction of starch metabolism, that is, synthesis through gluconeogenesis and remobilization through glycolysis, may account for the accumulation of hexoses and hexose‐phosphates observed under HL. However, investment into starch can come at the cost of protein synthesis when energy and C skeleton availability are restricted, that is, under HL‐Amb. Triose‐phosphates (TPs), that is, glyceraldehyde‐3‐phosphate and dihydroxyacetone‐phosphate, produced through the CBB cycle, can be exported from the chloroplast to sustain metabolic activity in other cell compartments (Johnson and Alric 2013). Hence, the higher levels observed for both intermediates, and for pyruvate, under HL‐CO_2_ but not HL‐Amb, as compared to the corresponding LL treatments, likely reflect that HL only promoted the export of TPs and further use in the cytosolic glycolytic pathway under CO_2_. Transfer of reducing power from the chloroplast to the TCA cycle in the mitochondria through the malate valve may instead substantially account for the respiratory rates measured under HL‐Amb and contribute to powering the CCM and to energy balancing in the chloroplast as previously suggested (Dao et al. 2022; Pfleger et al. 2025). The TCA cycle may not only contribute to ATP production, but also provide C skeletons for amino acid production (Sweetlove et al. 2010; Pfleger et al. 2025), hence influencing N assimilation. Indeed, *C. reinhardtii* has considerable plasticity in elemental composition, with C:N ratios measured from 5 to 26 depending upon growth conditions (Isanta‐Navarro et al. 2024). An induction of N metabolism, and especially of amino‐acid accumulation, was previously reported under HL, which was attributed to de novo synthesis rather than protein degradation (Davis et al. 2013). Accordingly, a clear accumulation was observed for most amino acids and other intermediates of the N metabolism in HL‐CO_2_, but not in HL‐Amb cells (Figure 6), which reflected their increased protein content (Figure 2E), as previously reported (Pfleger et al. 2025). Hence, the increased availability of C skeletons under HL‐CO_2_ likely promoted N assimilation, which became constrained under C_i_ limitation.

HL accumulated proteins associated with N metabolism comprised enzymes involved in methionine synthesis, including the cystathionine gamma‐synthase CGS1 (Cre03.g144627) and the cobalamin‐dependent methionine synthase METE1 (Cre03.g180750), and a methylthioribulose 1‐phosphate dehydratase (DEP, Cre01.g016528), putatively involved in methionine salvage from polyamine synthesis (Figure S4). The methionine salvage pathway is universally used to regenerate methionine from methylthioadenosine (MTA), a by‐product of polyamine synthesis generated during the aminopropylation of putrescine into spermidine and of spermidine into spermine (Sekowska et al. 2018). Accordingly, both methionine and putrescine accumulated under HL (Figure 6). Putrescine is the most abundant endogenous polyamine in *C. reinhardtii* (Lin and Lin 2019) and was shown to be involved in N metabolism and storage, but also in promoting cell cycle progression (Theiss et al. 2002). Hence, the synergistic effects of light and CO_2_ on putrescine accumulation are consistent with faster growth.

### 4.6 The Amino Acid Metabolism Under Ambient CO_2_ Conditions Likely Reflected Higher Occurrence of Anaplerotic Reactions and Photorespiration

Alanine and lysine were the only amino acids less abundant in HL‐Amb compared to LL‐Amb cells. Alanine can support pyruvate formation via alanine aminotransferase (AAT1; Cre10.g451950), which is coded by a low CO_2_‐inducible gene and is important in photorespiration (Chen et al. 1996). Alanine is also the most abundant amino acid of both small and large subunits of RuBisCO, constituting 15.7% and 12.2% of total amino acids, respectively (Bateman et al. 2025), possibly explaining why it was depleted in HL‐Amb cells. Proteins involved in N metabolism that accumulated under Amb compared to CO_2_ include carbamoyl phosphate synthetase, CMP1 (Cre08.g358580), which directly connects C_i_ fixation to nitrogen assimilation, and glutamate dehydrogenases, GDH1 under HL and LL (Cre09.g388800) and GDH2 under HL (Cre05.g232150; Figure S4). It has been shown that GDH1 is important in N assimilation from ammonium, along with an aspartate transaminase, for example, AST3 (Cre02.g097900), and may contribute to the re‐assimilation of ammonia produced through photorespiration in the mitochondria (Dao et al. 2025). In contrast, GDH2 may play anaplerotic roles via conversion of glutamate into α‐ketoglutarate and ammonia (Moyano et al. 1995) to fuel the TCA cycle. Under Amb conditions, amino acid remobilization may support mitochondrial ATP production, potentially explaining why HL‐induced increases in TCA cycle intermediates were not accompanied by a corresponding rise in amino acid levels compared to LL. Exceptions of N metabolites that increased in HL‐Amb cells were N‐acetyl‐L‐glutamate (NAG), which is formed prior to the rate‐limiting step in arginine synthesis via the NAG kinase, as regulated by the nitrogen sensing PII protein, GLB1 (Vlasova et al. 2023), O‐acetyl‐serine, a precursor of cysteine and glycine, potentially produced through photorespiration.

Extracellular glycolate concentration was lowest under HL‐CO_2_ and highest under Amb conditions (Figure S7). Glycolate release by *C. reinhardtii* had previously been reported under photoautotrophic conditions and attributed to its production through photorespiration (Moroney et al. 1986) in line with the previously reported accumulation of some of the enzymes involved in this pathway, such as the glycerate kinase GLYK1 (Cre12.g542300) and subunits of the glycine decarboxylase complex GCSH1 (Cre06.g253350) and GCSP1 (Cre12.g534800; Pfleger et al. 2025), overall supporting that photorespiration was relevant to the C to N ratio of Amb cells.

### 4.7 High Light Promoted Glycerol Excretion as a Possible Energy Dissipation Mechanism

Extracellular metabolite profiles generally showed very low metabolite abundances, indicating that the majority of metabolites were not excreted into the media and, additionally, that cold‐quenching with 60% methanol, to immediately stop enzymatic reactions, did not lead to substantial cellular leakage, as previously reported (Lee and Fiehn 2008). Out of the metabolites found in the media, glycerol was by far the most prominent and accumulated both intra‐ and extracellularly under HL (Figure 7), whereby the total amount of glycerol released largely exceeded that retained within the cell. *C. reinhardtii* was reported to excrete complex sugars, such as sorbitol and glycerol, into the media, especially when exposed to stressful conditions (e.g., salinity stress, HL). Under such conditions, glycerol production may contribute to dissipating excess reducing equivalents and may represent a significant proportion of fixed carbon (Davis et al. 2013; Demmig‐Adams et al. 2017). Glycerol can be produced from DHAP, through conversion to glycerol‐3‐phosphate, consuming NAD(P)H. In *C. reinhardtii* , this two‐step process can be catalyzed by a bifunctional enzyme with glycerol‐3‐phosphate dehydrogenase and phosphatase activities in the chloroplast (Morales‐Sánchez et al. 2017). In conclusion, the majority of glycerol was released into the surrounding media under HL, which suggests that it may act as a strategy to consume excess reducing equivalents, hence lowering Y[NA], and to maintain cellular redox balance.

## 5 Conclusions

HL‐CO_2_ increased photosynthetic rates that also powered a higher rate of respiration to promote growth, but HL cells became less reliant on mitochondrial ATP production than LL cells. As part of HL acclimation, cells reduced their antenna size, which has a significant cost in terms of N for protein synthesis, and instead HL‐CO_2_ cells diverted resources towards increasing RuBisCO contents, whereas HL‐Amb cells relied more on dissipating excess light energy. Further, donor side limitation was alleviated under elevated CO_2_, enabling higher PSI electron flow, supported by increased Cyt *b*_*6*_ *f* abundance. Metabolite profiling showed the highest abundance of primary metabolites under HL‐CO_2_, whereas the CO_2_ effect was evident also under LL, with C_i_‐dependent photoacclimation influencing the interplay between the TCA cycle and N assimilation. Among extracellular metabolites, glycerol was strongly excreted under HL, supporting its involvement as an energy sink, whereas glycolate increased particularly under Amb, indicative of increased photorespiration. Together, *C. reinhardtii* acclimates to sub‐saturating and saturating light through tightly coordinated adjustments of the light reactions of photosynthesis, whereas high CO_2_ availability promotes N assimilation for growth and efficient metabolism.

## Author Contributions

T.R. and A.P. planned and designed the research; T.R. contributed resources. A.P., E.A., and T.R. conducted the experiments; A.P., E.A., and T.R. analyzed and interpreted the data; A.P., E.A., and T.R. drafted, revised, and edited the manuscript.

## Supporting information


**Figure S1:** Influence of CO_2_ availability and light intensity on O_2_ production, consumption, and production to consumption ratio.
**Figure S2:** Influence of growth conditions on the redox state of photosystem I reaction center chlorophyll (P700).
**Figure S3:** Characterization of β‐carotene isomers via DAD‐HPLC.
**Figure S4:** Influence of CO_2_ availability and light intensity on proteins associated with amino acid metabolism.
**Figure S5:** Influence of CO_2_ availability and light intensity on the accumulation of proteins involved in redox homeostasis.
**Figure S6:** Influence of CO_2_ availability and light intensity on the number of differentially accumulated primary metabolites.
**Figure S7:** Influence of CO_2_ availability and light intensity on extracellular glycolate accumulation.

**Table S1:** Proteomic data re‐analyzed from Pfleger et al. (2025).
**Table S2:** Statistical evaluation of the proteomic data.
**Table S3:** Metabolite profiling dataset.
**Table S4:** Statistical evaluation of the metabolite profiling data.

## Data Availability

The data that supports the findings of this study are available in the Supporting Information material of this article.
