# Supplementary material for: CO2 ‐Dependent Promotion of Photosynthesis Drives Metabolic Photoacclimation in Chlamydomonas reinhardtii
Source: Physiol Plant. 2025 Aug 27;177(5):e70461. doi: 10.1111/ppl.70461 (PMC12381912; doi:10.1111/ppl.70461)
Supplement: Supplementary file 1 — Figure S1: Influence of CO2 availability and light intensity on O2 production, consumption, and production to consumption ratio. Figure S2: Influence of growth conditions on the redox state of photosystem I reaction center chlorophyll (P700). Figure S3: Characterization of β‐carotene isomers via DAD‐HPLC. Figure S4: Influence of CO2 availability and light intensity on proteins associated with amino acid metabolism. Figure S5: Influence of CO2 availability and light intensity on the accumulation of proteins involved in redox homeostasis. Figure S6: Influence of CO2 availability and light intensity on the number of differentially accumulated primary metabolites. Figure S7: Influence of CO2 availability and light intensity on extracellular glycolate accumulation. [file PPL-177-e70461-s001.pdf]

CO_2-dependent promotion of photosynthesis drives metabolic photoacclimation in *Chlamydomonas reinhardtii.*

Ana Pfleger, Erwann Arc, Thomas Roach

Supplementary figures

Figure S1. Influence of CO_2 availability and light intensity on O_2 production, consumption and production to consumption ratio.

Figure S2. Influence of growth conditions on the redox state of photosystem I reaction center chlorophyll (P700).

Figure S3. Characterisation of β-carotene isomers via DAD-HPLC.

Figure S4. Influence of CO_2 availability and light intensity on proteins associated with amino acid metabolism.

Figure S5. Influence of CO_2 availability and light intensity on the accumulation of proteins involved in redox homeostasis.

Figure S6. Influence of CO_2 availability and light intensity on the number of differentially accumulated primary metabolites.

Figure S7. Influence of CO_2 availability and light intensity on extracellular glycolate accumulation.

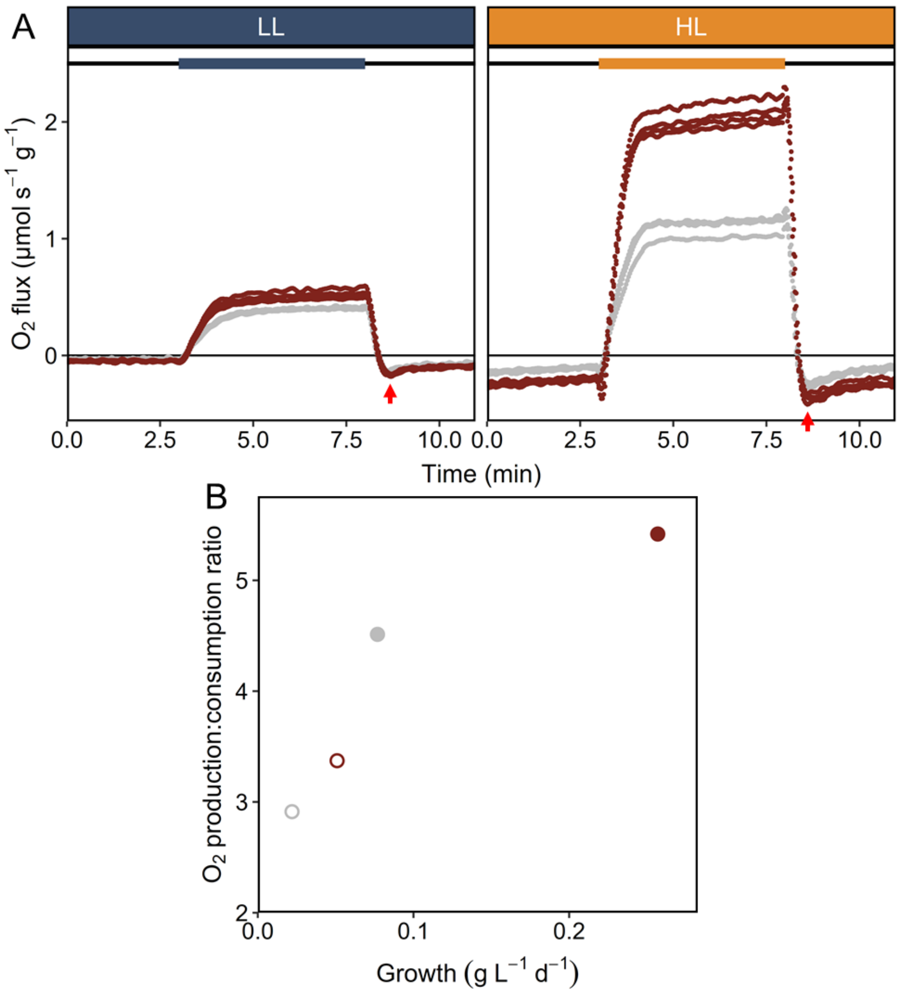

Figure S1. Influence of CO_2 availability and light intensity on O_2 production, consumption and production to consumption ratio. Cells were grown under low light (LL, 50 µmol m^{-2} s^{-1}, open symbols) or high light (HL, 500 µmol m^{-2} s^{-1}, closed symbols), with 0.04% (grey) or 2 % CO_2 (red). (A) O_2 flux measurements using the Photobiology (PB) module of a NextGen-O2k high resolution respirometer (Oroboros instruments). O_2 fluxes were determined at the light intensity's cells were grown under. Cells were initially kept in the dark to measure dark respiration, after which the light was turned on for 5 min to determine the net O_2 production. The peak of O_2 consumption observed about 30 sec after switching off the light (red arrows) was used as a measure of light enhanced dark respiration (LEDR). Oxygen fluxes were determined as the time derivative of the O_2 concentration, corrected for the instrumental background and normalised to the dry biomass (n= 4). (B) Relationship between growth and the ratio of gross O_2 production to maximum O_2 consumption immediately after switching off the light (used as a marker for light-enhanced dark respiration) under each culture condition.

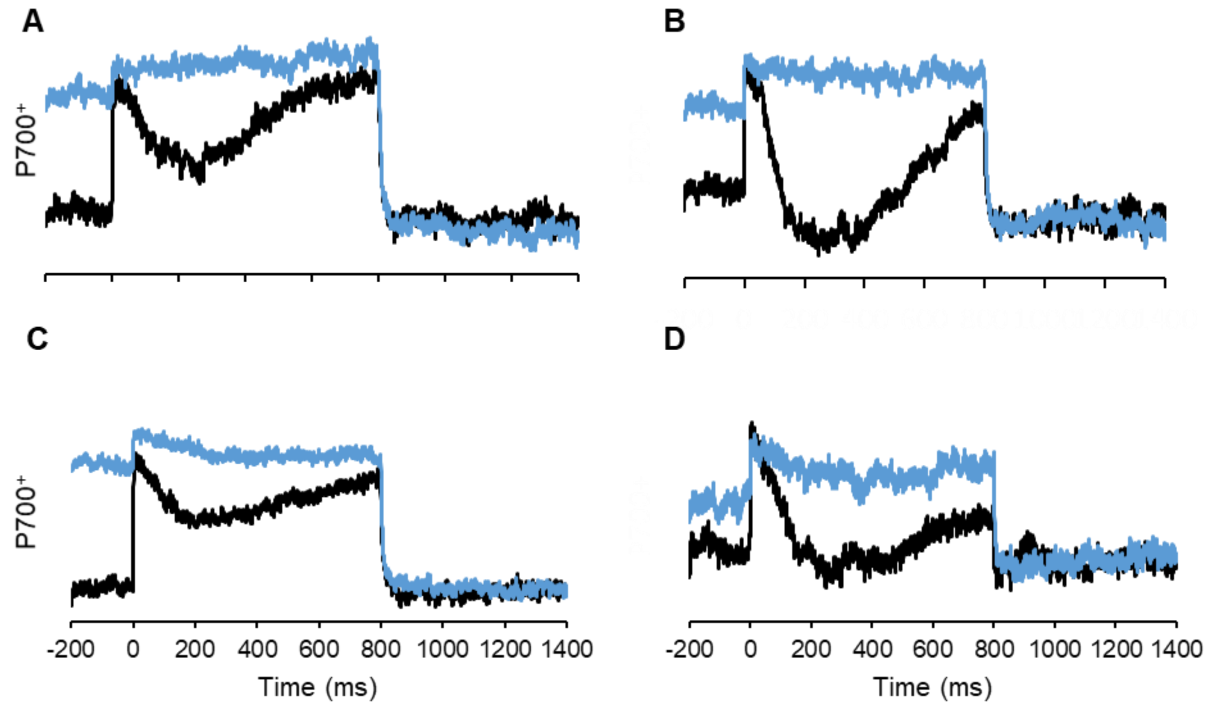

Figure S2. Influence of growth conditions on the redox state of photosystem I reaction center chlorophyll (P700). Cultures were grown in (A, B) 0.04 % or (C, D) 2 % CO_2, under continuous (A, C) low light (LL, 50 µmol m^{-2} s^{-1}) or (B, D) high light (HL, 500 µmol m^{-2} s^{-1}). Cells were probed with an 800 ms saturating pulse, starting at 0 ms, pre-treated with LL (black) or HL (blue), with 800 – 1400 ms under darkness. Traces show the level of oxidised P700 ($P700^{+}$), which is 0 in darkness. Typical measurements of one of the replicates is shown.

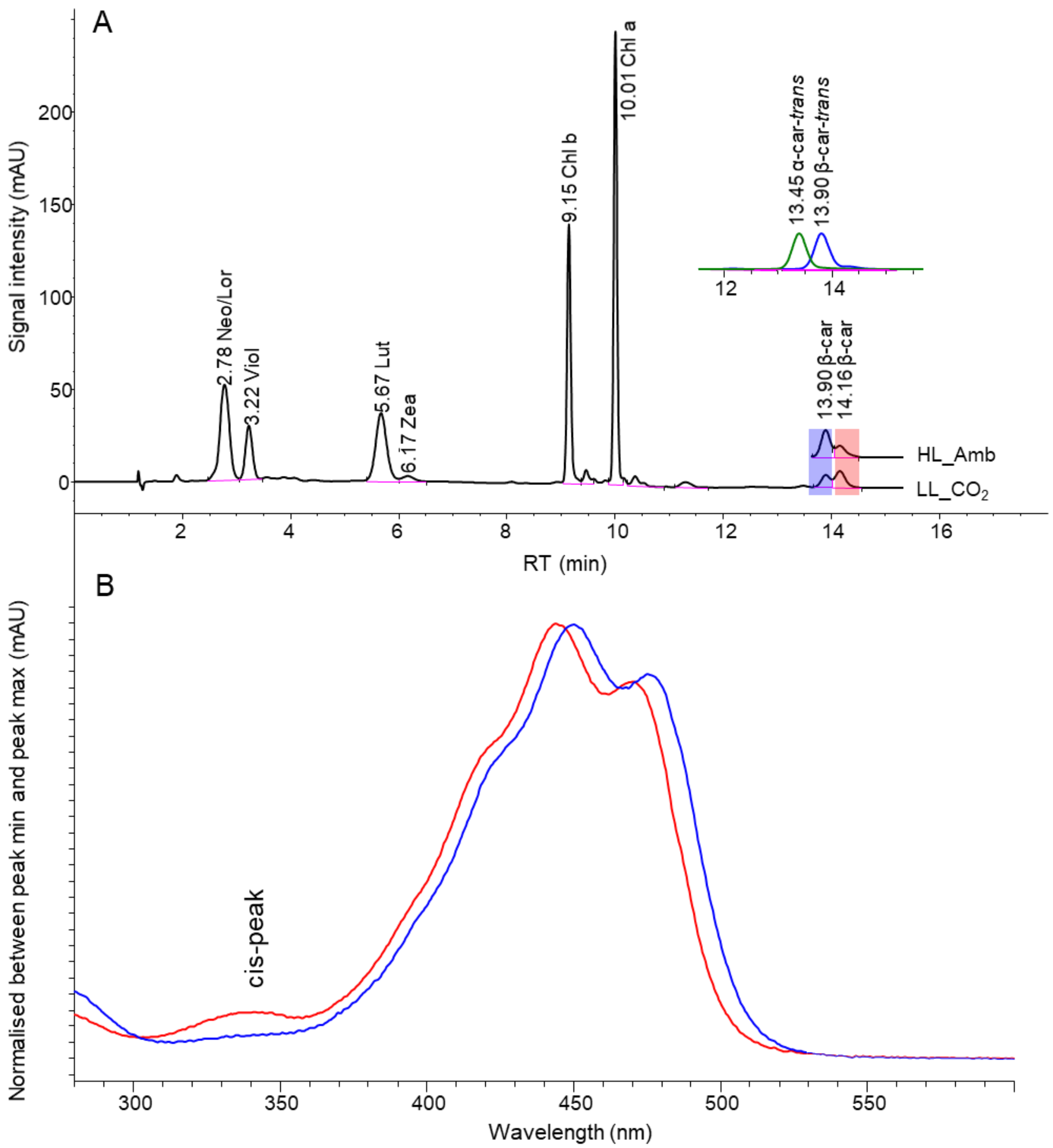

Figure S3. Characterisation of β-carotene (β-car) isomers via DAD-HPLC. (A) Chromatogram of total pigments at 440 nm absorbance of a typical LL-CO_2 sample. Pigments corresponding to each peak are labelled above the bar with respective retention times (RT). A partial chromatogram of *trans* α-car (green) and *trans* β-car (blue) standards, as well as a typical HL-Amb sample between RT of 13.5-14.5 min (during elution of β-car) are superimposed above. The two putative β-car isomers have been shaded blue and red. (B) Absorption spectra of the blue and red shaded peaks in (A) of LL-CO_2 sample, corresponding with RT of the *trans* β-car standard (blue spectra) and putative *cis* (red spectra) isomers of β-car, respectively, showing a hypsochromic shift and a"*cis*-peak" at 340 nm in the later-eluting β-car peak.

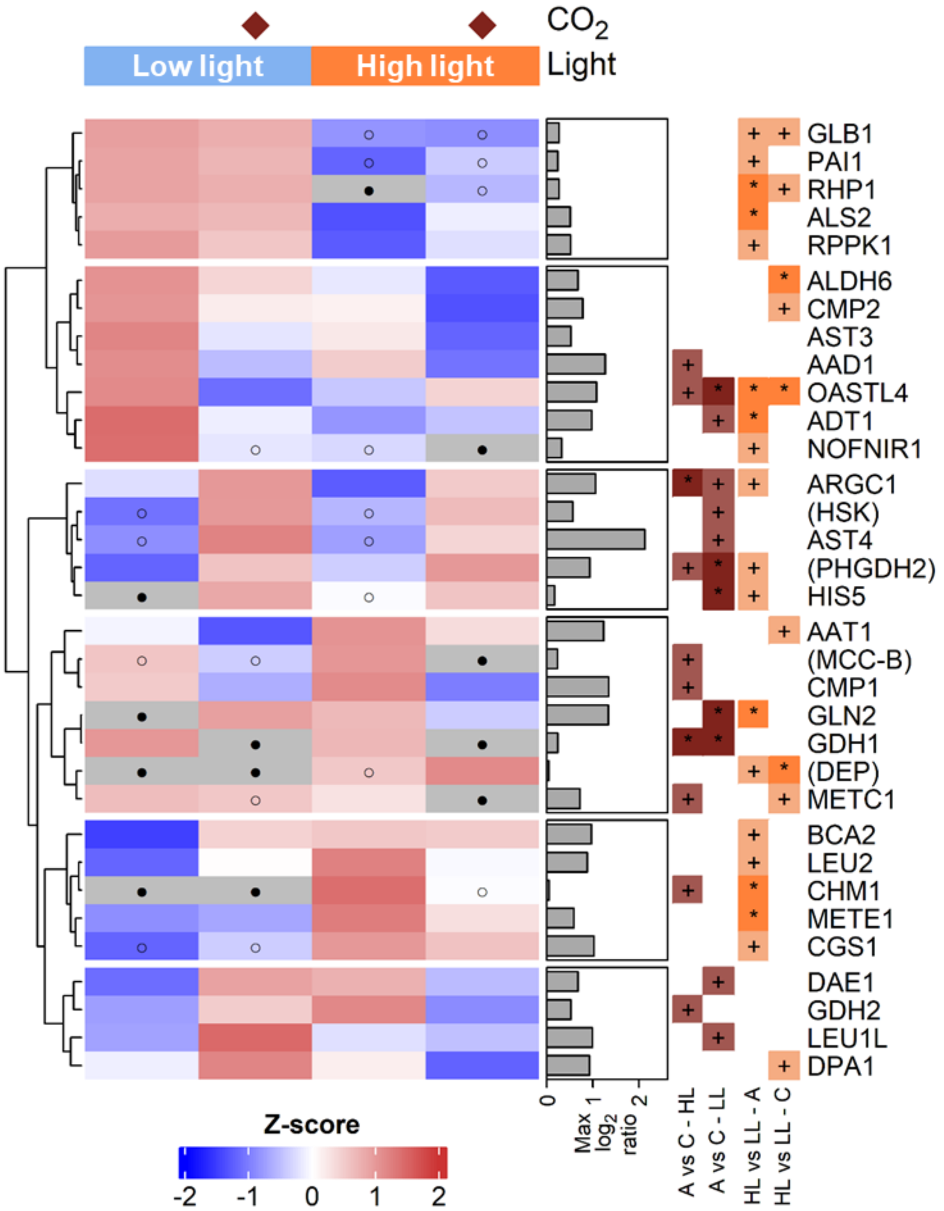

Figure S4: Influence of CO_2 availability and light intensity on proteins associated with amino acid metabolism. Cells were grown under low light (50 µmol m^{-2} s^{-1}, LL) or high light (500 µmol m^{-2} s^{-1}, HL), with 0.04% (A) or 2 % CO_2 (C, diamonds above the heatmap). Z-scores of mean protein relative abundances (*n*= 3) are shown on a colour scale from blue to red for nitrogen metabolism related proteins showing differential accumulation in at least one pairwise comparison between growth conditions (FDR adjusted *p*-value < 0.05). Proteins were hierarchically clustered using the Euclidean distance and Ward's clustering method. Grey bars (right) denote min-max log2 fold differences (not considering conditions under which proteins were not detected). * and + on the right side respectively denote FDR-adjusted or non-adjusted *p*-value below 0.05 for the pairwise comparison indicated at the bottom. Protein annotations are provided in Supplementary Table S1.

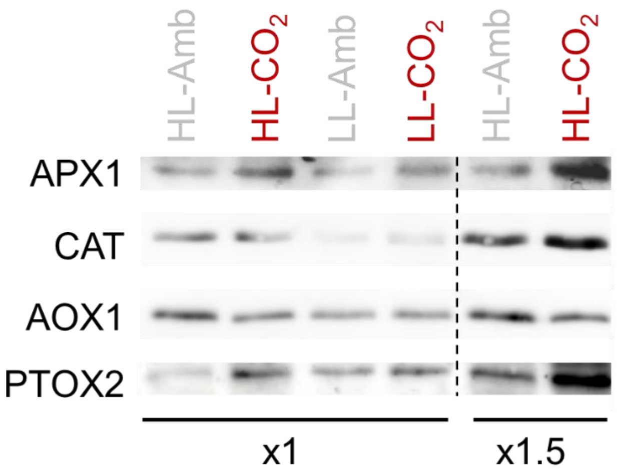

Figure S5: Influence of CO_2 availability and light intensity on the accumulation of proteins involved in redox homeostasis. Cells were grown under low light (LL, 50 µmol m^{-2} s^{-1}) or high light (HL, 500 µmol m^{-2} s^{-1}), with 0.04% (grey) or 2 % CO_2 (red). Proteins were detected by western blot using antibodies against ascorbate peroxidase 1 (APX1), catalase (CAT), alternative oxidase 1 (AOX1) and plastid terminal oxidase 2 (PTOX2). All samples were loaded relative to equal culture optical density at 720 nm (x1), with an additional 50 % sample loading for HL cultures only (x1.5) to reveal if saturation point of the blot had been reached in x1.

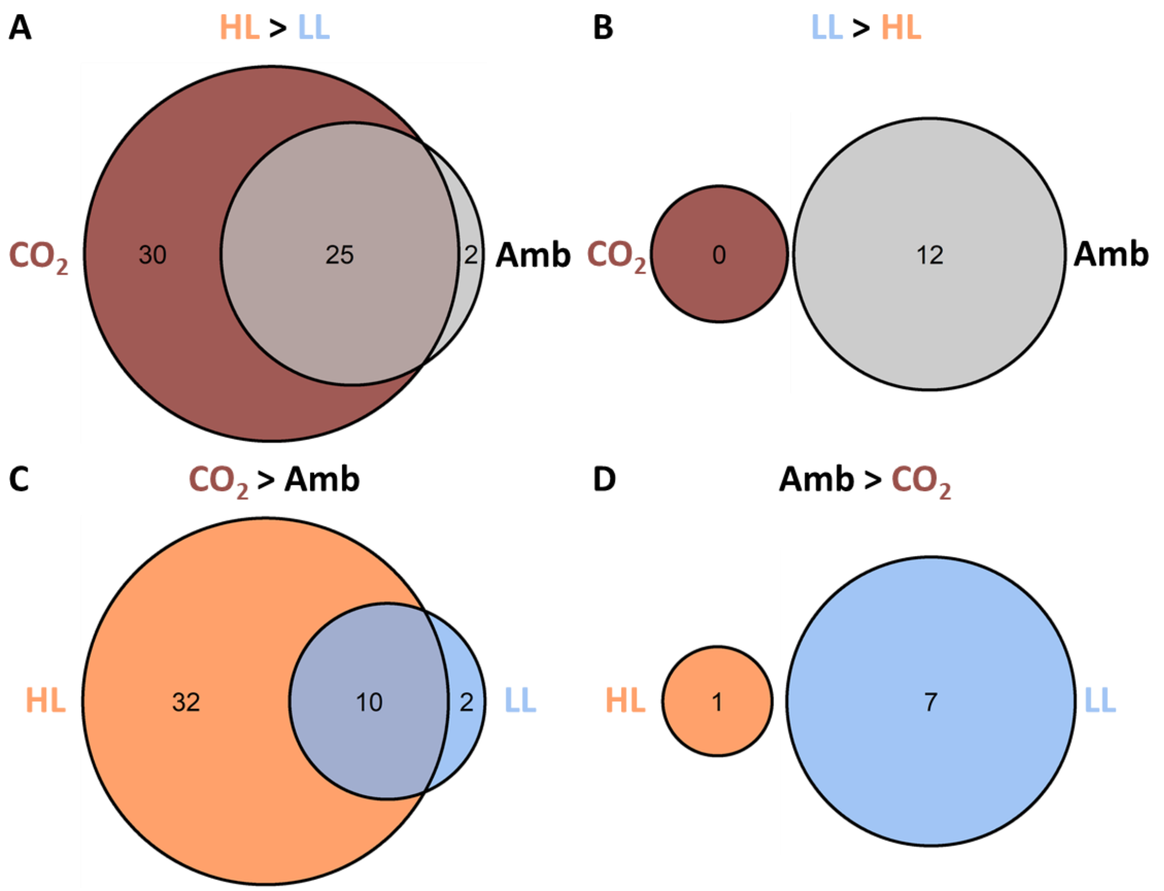

Figure S6: Influence of CO_2 availability and light intensity on the number of differentially accumulated primary metabolites. Cells were grown under low light (LL, 50 µmol m^{-2} s^{-1}) or high light (HL, 500 µmol m^{-2} s^{-1}), with 0.04% (Amb, grey) or 2 % CO_2 (CO_2, dark red). Venn diagrams display the number of primary metabolites found differentially accumulated (FDR-adjusted *p*-value < 0.05) either between HL and LL under Amb or CO_2 (A & B), or between CO_2 and Amb under HL or LL (C & D). Overlaps between the circles depict metabolites showing differential accumulation in both pairwise comparisons considered. Refer to Supplementary Table S3 for further details.

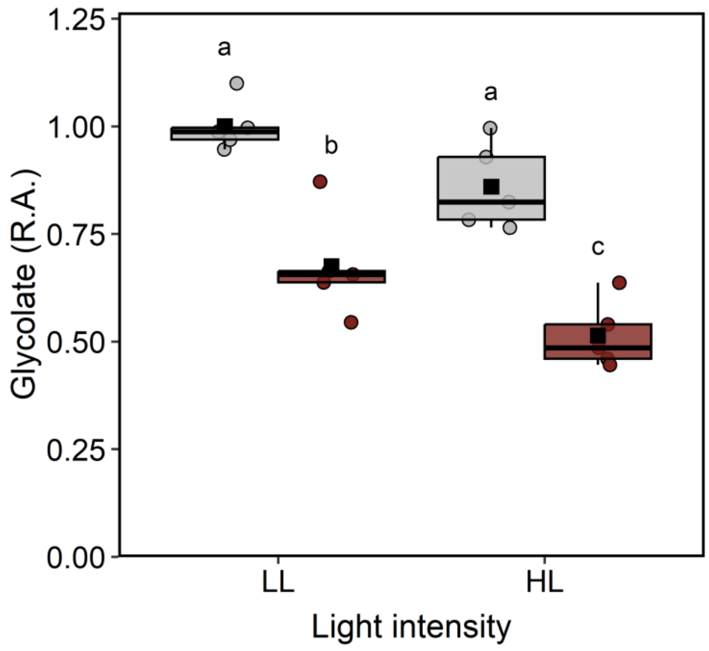

Figure S7: Influence of CO_2 availability and light intensity on extracellular glycolate accumulation. Cells were grown under low light (LL, 50 µmol m^{-2} s^{-1}) or high light (HL, 500 µmol m^{-2} s^{-1}), with 0.04% (grey) or 2 % CO_2 (dark red). Glycolate content in the growth medium was determined by GC-MS after quenching with 60 % methanol and expressed as a relative abundance per dry cell biomass. Box plots show medians and the 25th and 75th percentiles, dots represent individual data points and black squares correspond to the means (n= 5). Different letters denote significant differences (FDR adjusted p-value < 0.05).
